# Mechanistic insights on Bi-potentiodynamic control towards atomistic synthesis of electrocatalysts for hydrogen evolution reaction

**DOI:** 10.1038/s41598-023-43301-9

**Published:** 2023-09-30

**Authors:** Rohit Ranjan Srivastava, Divyansh Gautam, Rajib Sahu, P. K. Shukla, Bratindranath Mukherjee, Anchal Srivastava

**Affiliations:** 1 Department of Physics, Institute of Science, Banaras Hindu University, Varanasi, 221005 India; 2 Department of Metallurgical Engineering, Indian Institute of Technology-BHU, Varanasi, 221005 India; 3 Max-Planck-Institut für Eisenforschung, 40237 Düsseldorf, Germany; 4 Vindhya Institute of Technology and Science, Satna, MP 485001 India

**Keywords:** Materials science, Nanoscience and technology

## Abstract

Herein, electrochemically assisted dissolution-deposition (EADD) is utilized over a three-electrode assembly to prepare an electrocatalyst for hydrogen evolution reaction (HER). Cyclic voltammetry is performed to yield atomistic loading of platinum (Pt) over SnS_2_ nanostructures via Pt dissolution from the counter electrode (CE). Astonishingly, the working electrode (WE) swept at 50 mV/s is found to compel Pt CE to experience 1000–3000 mV/s. The effect of different potential scan rates at the WE have provided insight into the change in Pt dissolution and its deposition behaviour over SnS_2_ in three electrode assembly. However, uncontrolled overpotentials at CE in a three-electrode assembly made Pt dissolution-deposition behavior complex. Here, for the first time, we have demonstrated bi-potentiodynamic control for dissolution deposition of Pt in four-electrode assembly over Nickel (Ni) foam. The dual cyclic voltammetry is applied to achieve better control and efficiency of the EADD process, engendering it as a pragmatically versatile and scalable synthesis technique.

## Introduction

Platinum (Pt), established as a highly active catalyst for hydrogen evolution reaction (HER), offers challenges of cost and scarcity, which eventually hinders its large-scale applications^1^. HER catalysts have improved significantly over the last few decades, but most of them are still inferior to noble metals. Furthermore, it is also important to improve the electrochemical stability of HER catalysts by loading them with noble metals^2,3^. In this perspective, downsizing the noble metals from bulk to nanoclusters or isolated single atoms is an effective approach for utilizing maximum atom efficiency and catalytic activity^4^. Therefore, developing a cost-effective, trace amount atomic layer deposition of Pt-based electrocatalysts is still a formidable challenge and an ultimate goal. In recent years, significant progress has been made to decorate Pt atoms on various substrates using chemical vapor deposition, atomic layer deposition, pyrolysis, vacancies/defects-based immobilization strategies, hydrothermal methods, etc.^5^. However, most of these synthesis methods deliver the bulk deposition over the substrate and are expensive to upscale. Bulk deposition of active elements decreases the number of electrochemically active sites on the catalyst surface. The electrochemical deposition technique has emerged as a universal method for synthesizing electrocatalysts with trace amounts of noble metals^5^. Moreover, this technique has gained wide attention due to its characteristics such as (i) tunability of particle size by changing the deposition parameters, (ii) deposition of metal over the surface of a substrate, and (iii) providing a large number of active surface sites^6^. As a result, electrochemical deposition emerged as a surface synthesis method for catalytic applications. Recent studies show that the trace amount of Pt dissolution takes place in the HER process, which was not commonly noticed^7^. In this work, we propose that Pt dissolution during HER can be put into the perspective of developing a synthesis route for controlled Pt deposition from the counter electrode (CE) into various substrates (2D materials such as Graphene, MoS_2_, WS_2_, SnS_2_, etc.) used as working electrode (WE). However, building a synthesis technique of Pt incorporation into various materials requires more understanding of the dissolution-deposition behaviour from Pt_CE_^8^. Pt dissolution from CE during HER process may alter the electrochemical environment on WE, such as (i) instigates a particular faradic reaction while suppressing the others, (ii) change in open circuit potentials (OCP) of both the electrodes etc. Interestingly, we have found in the present study that the scan rate experienced by CE is not the same as that applied over WE, instead it is 20–60 times higher, thereby making the Pt dissolution from CE peculiar and challenging to understand.

Anchoring Pt atoms on a specific anchoring substrate material (used as WE) depends on the substrate’s surface properties such as morphology, structure, and chemical composition, which profoundly regulate the active sites on the electrode. Therefore, designing an electrochemically active, stable, and cost-effective electrocatalyst using ultra-low amount of Pt is still challenging. During the last few years, layered metal dichalcogenides (LMDs such as MoS_2_, WS_2_, MoSe_2_, etc.) have gained wide attention owing to their peculiar properties such as high surface to volume ratio, catalytic activity and stability. Though, most sulfides have low carrier density, less active sites and poor conductivity. But, these limitations can be overcome by decorating LMDs with a modest quantity of noble metals using various methods, which can enable faster charge transfer during catalysis^9–11^. Among these LMDs, in tin disulfide (SnS_2_), the central tin (Sn) atom is covalently bonded to six other sulfur (S) atoms at octahedral sites, attached by weak van der Waal forces among the adjacent layers^12,13^.

Herein, Electrochemically Assisted Dissolution-Deposition (EADD) process has been demonstrated as a unique synthesis technique for atomistic or subnanometer scale Pt deposition over a substrate. The focus of the present work is to demonstrate atomic deposition of Pt over hydrothermally synthesized 2D SnS_2_ nanostructures and Ni foam using a bi-potentiodynamically controlled dissolution deposition technique. Moreover, prospectives of the dissolution-deposition behavior of Pt are put forward to understand and control the EADD process. After realizing the limitations of the EADD synthesis in a three-electrode configuration, an insight into the four-electrode assembly for controlled atomistic deposition of Pt over Ni foam has been provided for the first time. This proposed four-electrode configuration for EADD not only overcomes the challenges of uncontrolled dissolution-deposition encountered in a three-electrode assembly but also opens up enormous possibilities for the development of a versatile atomistic deposition technique to obtain novel catalysts for a variety of applications such as HER, fuel cells and other scientific and industrials applications.

## Methods

### Materials

In this work, all chemicals were of analytical grade and used without further purification. Tin tetrachloride pentahydrate (SnCl_4_·5H_2_O) and Thioacetamide (C_2_H_5_NS) were purchased from Molychem, India and SRL India, respectively. Nickel foam was purchased from china. H_2_SO_4_ was used for preparing the electrolyte solution (0.5 M) and purchased from Molychem India. KCl was purchased from Molychem, India and used in reference electrode. Nafion binder was purchased from Sigma Aldrich. Nafion Film and Graphite paper (99% pure) were purchased from Sigma Aldrich and Xiamen Tob new energy technologies respectively. Deionized water was used for synthesis, electrochemical and cleaning purposes.

### Characterization

The high-resolution transmission electron microscopy (HRTEM) was conducted using FEI spherical aberration (C_s_) image corrected Titan Themis at an acceleration voltage of 300 kV. Scanning transmission electron microscopic (STEM) measurement was performed using a C_s_ probe corrected FEI Titan instrument. High Resolution scanning electron microscope (HR-SEM) image was captured using (NOVA NANO SEM 450, FEI, USA. X-ray photoelectron spectroscopy (XPS) was performed on PHI 5000 VersaProbe III using 200 W monochromated Al Kα radiation. Inductively coupled plasma mass spectrometry (ICP-MS) was analyzed using Agilent, 8900 ICP-MS Triple Quad.EADD and HER measurements were performed using a Single channel Corrtest potentiostat (CS350) and double channel Corrtest bipotentiostat (CS2350). X-ray Diffractometer (PANanlytical, U.K) using Cu-Kα radiation (α = 1.54178 Å) at a scanning rate of 1°s^−1^ ranging from 10° to 80° and Raman spectrophotometer (Renishaw, UK) were used to analyze the structural phase purities of as-synthesized SnS_2_ nanostructures with a laser excitation of 532 nm.

### Electrochemical measurements

All electrochemical measurements for HER have been performed on CS350 single-channel Corrtest potentiostat on a three-electrode system using graphite rod as counter electrode setup and Ag/AgCl (3 M KCl) as a reference electrode. For OCP measurements of Pt CE, another CS2350 double-channel Corrtest bipotentiostat was used to operate the three-electrode assembly. All HER measurements were conducted in N_2_ purged in 0.5 M H_2_SO_4_ (deaerated electrolyte). The sweep rate of 10 mV/s was used to report all voltammetric measurements. All the polarization curves and corresponding Tafel plots are shown without IR compensation however wherever required IR compensated values have been mentioned with percentage compensation. Reference electrode data was converted to RHE by using Nernst equation (V_RHE_ = V_Ag/AgCl_ + 0.0591 pH + V°_Ag/AgCl_). EIS spectra were performed at − 0.122 V_RHE_ from 10^5^ to 0.1 Hz with an amplitude of 10 mV. The accelerated stability test was performed in the potential range of − 0.472 V_RHE_ to 0.228 V_RHE_ with a sweep rate of 100 mV/s for 28 h. Prepared SnS_2__10 mV/s, SnS_2__50 mV/s, SnS_2__100 mV/s and SnS_2__200 mV/s from the EADD experiments were deployed as the standing electrodes for HER experiments.

Four-electrode assembly was also used in the current work operated using a bipotentiostat (CS2350). Thoroughly cleaned Ni foam was deployed as main WE (anchoring substrate electrode) of exposed area 0.32 cm^2^, while Polycrystalline Pt was used as slave WE (sacrificial electrode) with exposed area 1 cm^2^. Pt as an auxiliary electrode was used to complete the circuit while Ag/AgCl (3 M) was used as a reference electrode in N_2_ purged 0.5 M H_2_SO_4_ electrolyte. Main WE were swept in − 0.435 V_RHE_ to − 0.335 V_RHE_ at 50 mV/s for 4050 cycles while slave WE were swept between 0 to 1.8 V_RHE_ at 300 mV/s for 1350 cycles. Sampling frequency was kept the same for both WEs as 100 Hz to make it synchronous.

### Material synthesis

#### Hydrothermal synthesis of SnS_2_ nanostructures

Tin (IV) chloride pentahydrate (SnCl_4_·5H_2_O) and Thioacetamide (C_2_H_5_NS) were used as the precursor of Tin (Sn^4+^) and sulfur (S^2−^) source respectively. Tin (IV) chloride pentahydrate (~ 1.6 g) and Thioacetamide (~ 1.4 g) were dissolved in 60 ml of distilled water and mixed well under vigorous string for 120 min. The complete solution was transferred in Teflon-lined autoclave at 180 °C for 12 h. After the proposed reaction time, the autoclave is kept at room temperature until cooled to room temperature. A yellowish color solution was obtained which was further washed several times with distilled water and finally dried the sample in a vacuum at 80 °C for 6 h. It was further utilized for EADD synthesis as well as a catalyst for electrochemical evaluation for hydrogen evolution reaction.

#### Fabrication of WE for electrochemical measurements

EADD was performed using three-electrode system at a Single channel Corrtest potentiostat (CS350) where Working electrode as anchoring substrate electrode (ASE), Counter electrode as a sacrificial electrode (SE) and Ag/AgCl (3 M KCl) electrode was used as a Reference electrode. Typically, SnS_2_ ink (3 mg) was prepared using ethanol/water (3:1 ratio) solution using Nafion binder (40 μl; 5 wt%) and sonicate rigorously. The above-prepared solution was drop cast over a window of 1 cm^2^ of one surface of thoroughly cleaned graphite sheets. These standing electrodes having the other exposed surface of graphite was covered by Teflon tape to expose only SnS_2_ solely in the electrolyte. The polycrystalline Pt was used as CE. Before deploying Pt as the sacrificial electrode, it used to be thoroughly cleaned in piranha solution for every experiment. The exposed area of Pt was kept deliberately to be 1 cm^2^ being quite low in comparison to standard CE protocol (generally kept 6–7 times higher than that of WE area) primarily used for the electrochemical study.

Herein, we conducted cyclic voltammetry over the WE (SnS_2_) with the potential range of − 0.9 to − 0.2 V vs Ag/AgCl and simultaneously altering the Pt CE dissolution and its deposition over WE by changing the scan rates over WE (ν_we_). There are four different scan rates (ν_we_) 10 mV/s, 50 mV/s, 100 mV/s and 200 mV/s chosen deployed for 400, 2000, 4000 and 8000 cycles respectively. The exposure time of the electrochemical cell was kept constant (~ 15 h) to observe the dissolution-deposition phenomena with varying scan rates. After activation of ~ 15 h, all the samples were gently washed by DI water and then heated at 100 °C for 2 h.

## Results

**Figure 1:**
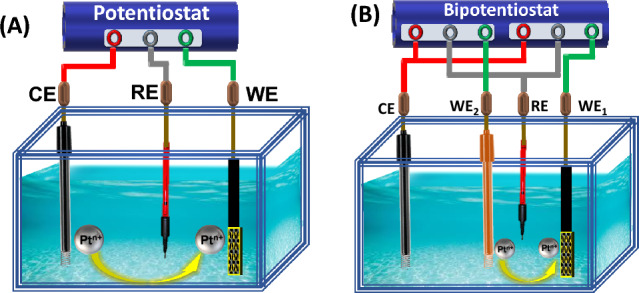
Schematic of electrochemically assisted dissolution-deposition setup using (**A**) Potentiostat and (**B**) Bi-potentiostat.

**Figure 2:**
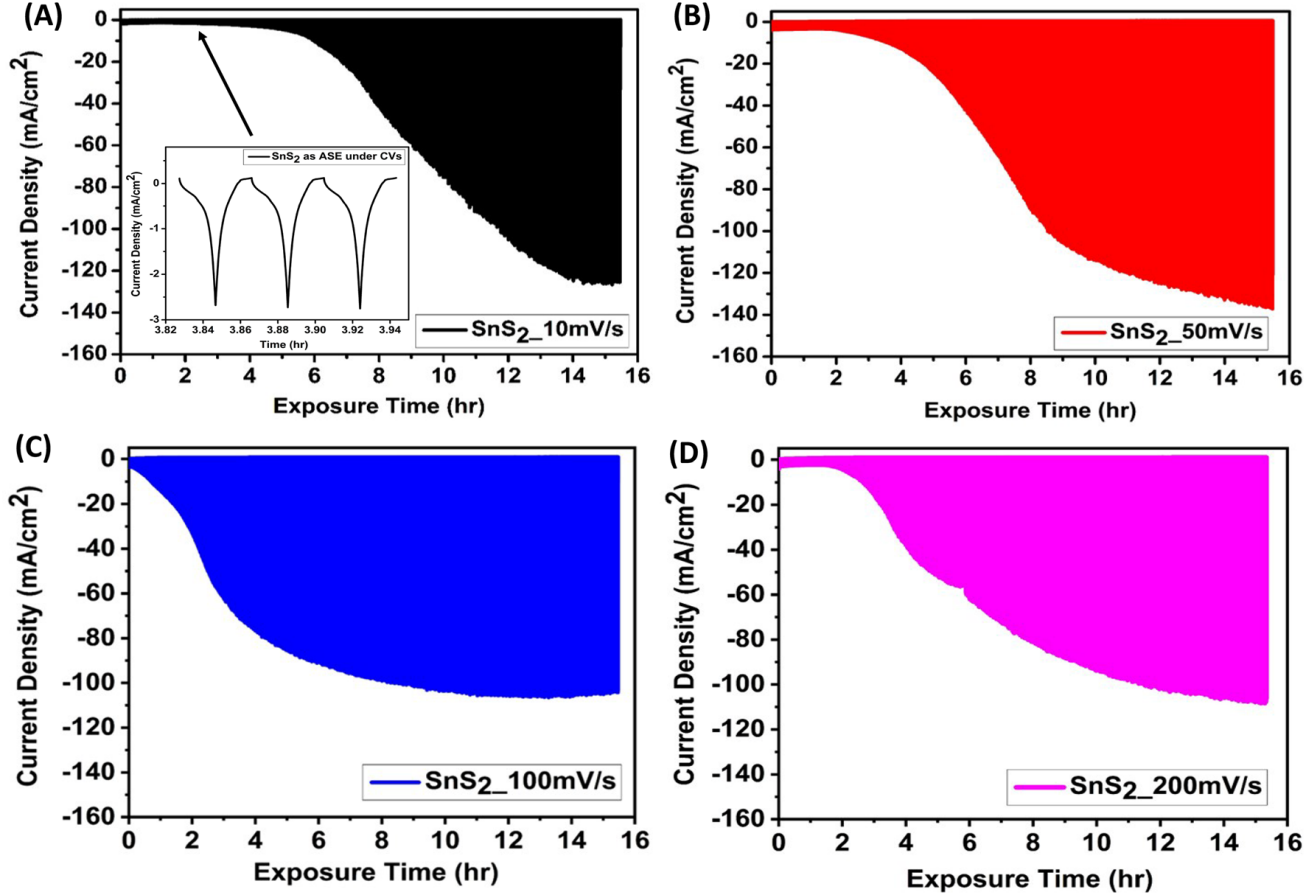
Current density vs time plots corresponding to Cyclic voltammetry conducted in acidic medium (0.5 M H_2_SO_4_) under potential range of lower potential (E_L_) = − 0.672 V_RHE_ to upper potential (E_U_)  = 0.028 V_RHE_ in a three electrode assembly at different scan rates: (**A**) 10 mV/s (SnS_2__10 mV/s), (**B**) 50 mV/s (SnS_2__50 mV/s), (**C**) 100 mV/s (SnS_2__100 mV/s) and (**D**) 200 mV/s (SnS_2__200 mV/s) for fixed exposure time (~ 15 h).

**Figure 3:**
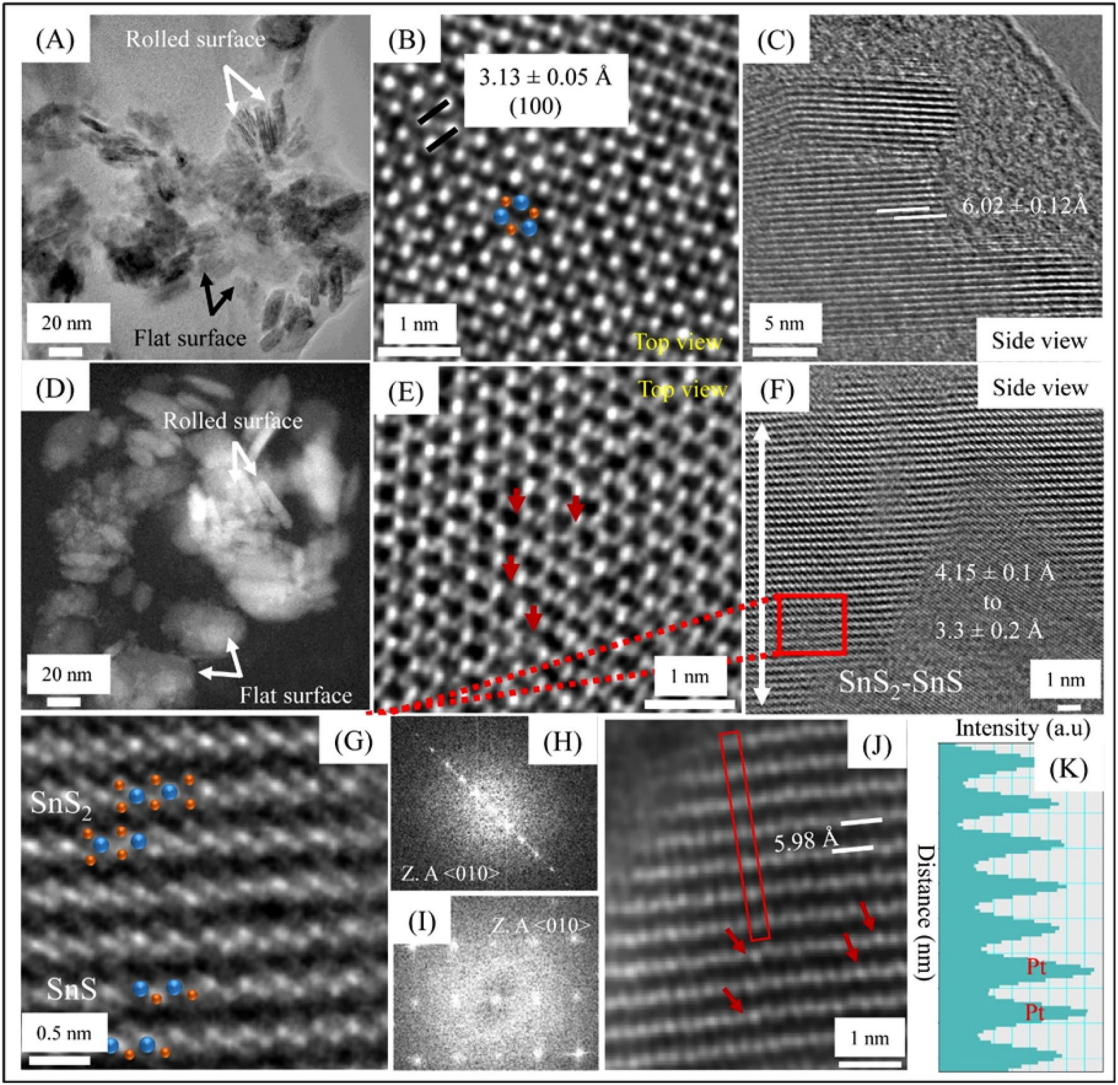
(**A**–**C**) HRTEM images of pristine SnS_2_. (**A**) Shows rolled and flat surface morphology in BF-TEM, (**B**,**C**) top and side view of SnS_2_. (**D**–**K**) HRTEM images after EADD synthesis of SnS_2_ @ 50 mV/s. (**D**) Shows rolled and flat morphology in annular dark feild (ADF) TEM, (**E**) the distorted phase of SnS_2_, (**F**) HRTEM micrograph showing SnS_2_–SnS heterostructure, (**G**) atomic arrangement of SnS_2_-SnS heterostructure, (**H**,**I**) FFTs images of SnS_2_ and SnS phases, (**J**,**K**) Pt distribution inside SnS_2_ layer.

It has been observed that Pt undergoes dissolution during oxygen reduction reaction (ORR) and oxygen evolution reaction (OER) in three-electrode assembly^14,15^. The kinetics of Pt dissolution depends on operational parameters such as the potential window, scan rate, the relative area of WE/CE, pH, presence of gaseous species, electrolyte composition and temperature^16^. Pt dissolution occurs at Pt_CE_ when WE are swept over a particular potential window in three electrode assembly as shown in Fig. 1A. Consequently, Pt ions coming into the bulk electrolyte get deposited over WE when the latter is subjected to reduction potentials of Pt/Pt^2+^ redox couple. To understand the insights of the dissolution-deposition process, cyclic voltammetry of SnS_2_ is conducted at four different scan rates (10, 50, 100 and 200 mV/s) over a fixed exposure time (~ 15 h) in 0.5 M H_2_SO_4_ (shown in Supplementary Fig. S1). The corresponding current vs. time plots are shown in Fig. 2. With the increase of the number of cycles, the current density is found to augment for all the deployed scan rates. Initially, there is no increment until the dissolution starts, i.e., up to 0.5–0.6 V_RHE_ experienced by Pt CE as shown in Supplementary Fig. S3. After the onset of dissolution, the deposition is readily observed in Fig. 2A–D. The increase in current density with scan rates as well as the number of cycles suggests that the number of active sites is increasing due to Pt deposition over SnS_2_, which is evident in Fig. 3.

However, saturation in the current density observed for all the scan rates (after ~ 100–110 mA/cm^2^), that can be attributed to the redeposition of Pt ions present in the double layer on the Pt_CE_ surface^17,18^, reducing the overall extent of Pt deposition compared to extent of Pt dissolution as evidenced in ICP-MS (Supplementary Fig. S4). At faster scan rates, the time duration to migrate Pt ions from the electrical double layer to the bulk electrolyte via diffuse layer minimizes the diffusion of Pt ions in the bulk electrolyte, increasing the double layer Pt ion concentration, resulting in the increased redeposition kinetics. Moreover, the potential window wider than 0.8–1.8 V has been found to yield Pt nanoparticles on the Pt_CE_ surface^17,19^. Moreover, it has been noticed that when the working electrode (WE) is swept at 50 mV/s, the Pt_CE_ encounters 1000–3000 mV/s. Therefore, in this study, potential window and scan rates experienced by Pt_CE_ have been further explored to understand the dissolution behaviour.

Here, a bi-potentiostat was used to measure the open circuit potential (OCP) of Pt_CE_ (wrt RE) during EADD synthesis, wherein, WE were swept at 50 mV/s, to collect the potentials and scan rates over the CE. As shown in Supplementary Fig. S2, the collected OCP data of CE (V_CE_ vs. t wrt Ag/AgCl reference electrode) was used to obtain sweep potentials at CE, which were then converted wrt RHE using Nernst equation^20^. It is observed that CE experiences an upper potential limit of 2.0 V_RHE_, which increases to 2.6 V_RHE_ as the cathodic HER current increases over WE (SnS_2_). However, lower potential limit changes from 1.8 to 0.55 V_RHE_ due to anodic oxidation of SnS_2_ over WE (Supplementary Fig. S3). The current density vs. potential (J–V) response of Pt_CE_ exhibits relatively higher scan rates (1000–3000 mV/s) compared to WE (50 mV/s), from the 1st to 15th hour, as shown in Table S1 (in Supplementary Information). Therefore, further increasing the scan rates over WE will result in even higher and higher values of scan rates over CE in three-electrode assembly. These findings suggest that faster scan rates instigate Pt redeposition kinetics and gradually dominate over intended dissolution kinetics^17,19^. As shown in Supplementary Fig. S4, for SnS_2__10 mV/s, SnS_2__50 mV/s, SnS_2__100 mV/s and SnS_2__200 mV/s, the extent of Pt dissolution was doubly confirmed by ICP-MS measurements of electrolyte, which shows dissolved Pt of 0.543, 0.445, 0.385 and 0.258 ppm and ICP-MS measurements of the deposited Pt over SnS_2_ WEs exhibit 0.041, 0.091, 0.098 and 0.045 ppm respectively. Thus, the amount of Pt deposited over WE is much less than the Pt dissolved from CE in the bulk electrolyte. This is on account of the formation of a hydrophobic gap, which may slow down the Pt deposition rate during HER^18,21^. Also, ICP-MS measurements reveal that the extent of Pt dissolution from CE decreases as the scan rates over WE increase (Supplementary Fig. S4A).

### Morphological and photophysical characterizations of electrodeposited Pt over defect rich SnS_2_

Pt deposition on SnS_2_ was further investigated for structural, elemental, compositional aspects using high-resolution transmission electron microscopy (HR-TEM) and X-ray photoelectron spectroscopy (XPS). HR-TEM images shown in Fig. 3 depict the morphology of atomically deposited Pt over SnS_2_. Figure 3A shows the bright-field TEM micrograph of pristine SnS_2_, which demonstrates rolled and flat morphology of SnS_2_ nanostructures. Figure 3B depicts a top view of a high-resolution HRTEM image displaying the trigonal structure of SnS_2_ with (100) plane oriented with a d-spacing of 3.13 ± 0.05 Å^11^. The side view of the HRTEM image as shown in Fig. 3C confirms that the interlayer van der Waal (vdW) spacing value is corresponding to the (001) plane of SnS_2_. However, after EADD synthesis of ~ 15 h, both rolled and flat surface morphology are visible in annular dark-field micrograph (Fig. 3D,E), revealing the defected trigonal structure due to sulfur (S) vacancies or defects. The HRTEM image (Fig. 3F) shows the formation of SnS_2_–SnS heterostructures as a result of the redox reaction during EADD synthesis. At the SnS_2_–SnS interface, the interlayer vdW distances inside the SnS region were observed as 3.3 ± 0.2 Å and that for SnS_2_ region as 4.15 ± 0.1 Å. This might be due to the S defects. Figure 3G shows the atomic arrangement of SnS_2_ and SnS, which corresponds to 1 T and orthorhombic phase, respectively. Fast Fourier Transform (FFT) was used to analyze the crystal structure in reciprocal space to confirm the existence of both SnS_2_ and SnS phase shown in Fig. 3H,I^22^. The Z (atomic number)-contrast HAADF STEM image and corresponding line profile from the rolled surface region (Fig. 3J,K), suggesting platinum (Pt) atoms replacing the tin (Sn) column. Elemental intensity distributions were mapped as shown in Supplementary Fig. S5. The elemental intensity distribution map indicates the distribution of Pt atomic cluster (1–2 nm) in SnS_2_.

**Figure 4:**
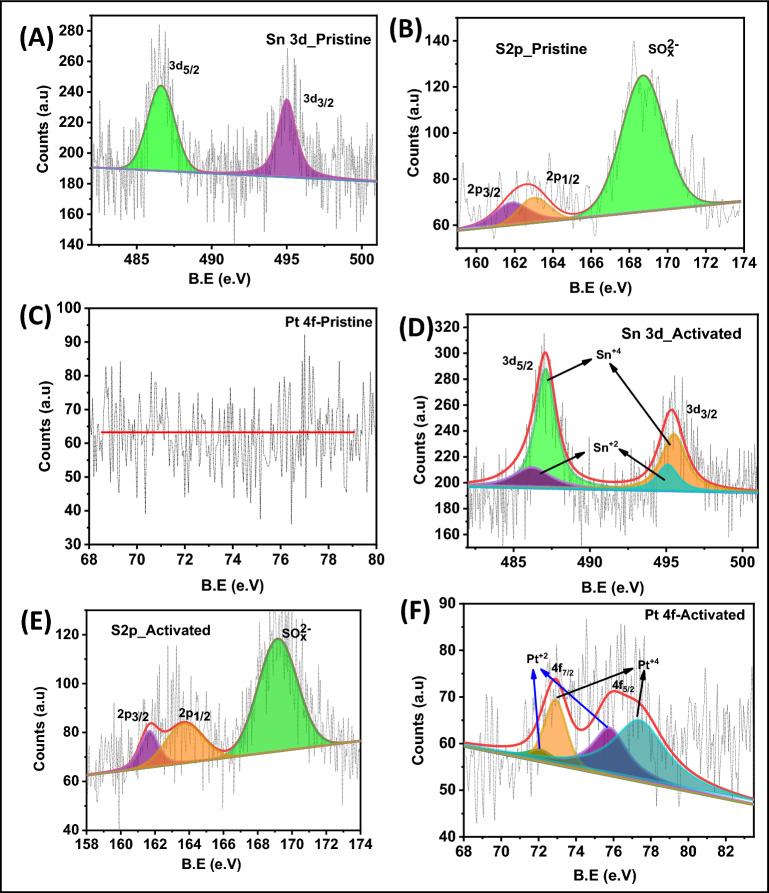
XPS spectra of (**A**) Sn (3d), (**B**) S (2p), (**C**) Pt (4f) of pristine SnS_2_ and (**D**–**F**) shows the XPS spectra of Sn (3d), S (2p) and Pt (4f) respectively after EADD synthesis.

To further explore the deposited Pt over SnS_2_, the elemental and surface chemical states were also investigated using XPS (Fig. 4). The XPS spectra of the pristine SnS_2_ are shown in Fig. 4A–C. As displayed in Fig. 4A, the Sn 3d peak is deconvoluted in two peaks ranging from 484 to 500 eV. The peaks at 486.5 eV and 495 eV correspond to 3d_5/2_ and 3d_3/2_, suggesting the Sn^4+^ oxidation state^11,23^. The XPS spectrum of S 2p is deconvoluted into three peaks ranging from 160 to 174 eV, as shown in Fig. 4B^11^. The peaks at 162 eV and 163 eV corresponds to 2p_3/2_ and 2p_1/2_, which suggest the presence of sulfur species such as terminal (S_2_^2−^, S^2−^) and apical (S^2−^) ligands in SnS_2_^11,24^. In Fig. 4C, there is no obvious peak observed for Pt 4f. After the EADD process, significant changes are observed, as shown in Fig. 3D–F. The Sn 3d and S 2p peaks in XPS spectrum (Fig. 4D,E) exhibits slightly oxidized states. In addition to this, there is an introduction of local defects during electrochemical synthesis. This confirms the phase transformation from SnS_2_ into SnS during EADD synthesis. Moreover, the enhanced Pt deposition on defect-rich SnS_2_ during the EADD process is further evaluated using XPS spectra, as shown in Fig. 4F. The Pt 4f is deconvoluted in two peaks ranging from 68 to 83 eV. The peaks at 72.1 eV, 72.9 eV and 75.8 eV, 77.4 eV corresponds to 4f_7/2_ and 4f_5/2_ of Pt^2+^ and Pt^4+^ respectively^10,25,26^. The presence of Pt^4+^ and Pt^2+^ explains that Pt-based clusters might be having a sulfur and oxygen environment.

#### Study of HER performance

**Figure 5:**
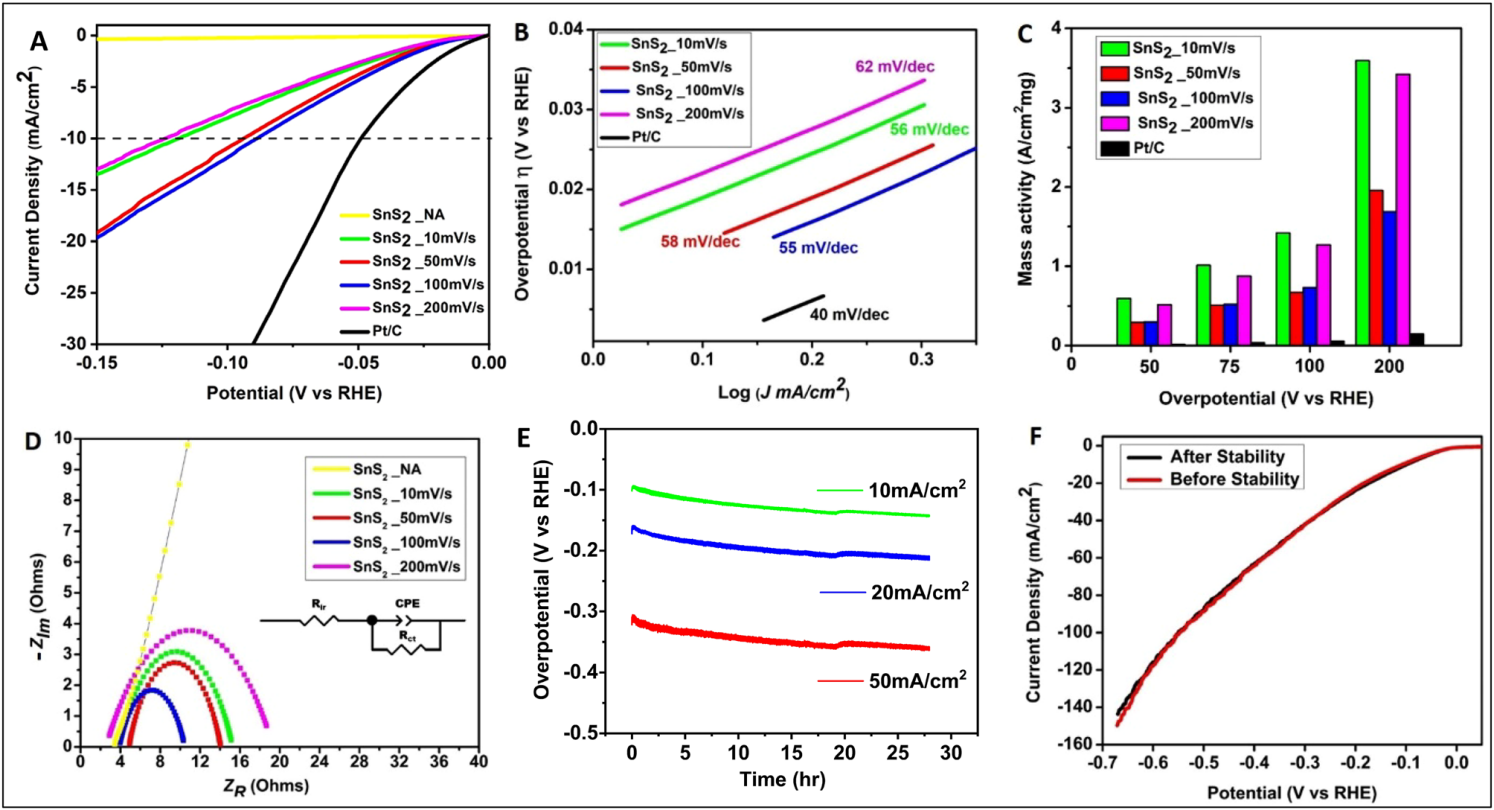
HER performance of various samples (**A**) shows polarisation curves, (**B**) shows corresponding Tafel plot, (**C**) represents mass activity plots corresponding to Pt loading over various samples. (**D**) Depicts Nyquist plots collected by Electrochemical Impedance Spectroscopy (EIS). (**E**) Shows J vs. t plots redrawn by Accelerated Stability Test (AST). (**F**) Shows polarisation curves collected before and after AST.

To study hydrogen evolution kinetics over the prepared SnS_2_-Pt catalyst, polarization curves were collected using linear sweep voltammetry (LSV) at 10 mV/s for all the samples, as shown in Fig. 5A. The measured onset potential (η_0_) and overpotential at 10 mA/cm^2^ of cathodic current density (η_10_) for pristine SnS_2_ are − 390 mV and − 826 mV respectively. Following Pt deposition over defect rich SnS_2_, the measured values of η_0_ is 14 mV, 4 mV, 4 mV, 17 mV and that of η_10_ is 112 mV, 87 mV, 83 mV, 119 mV for SnS_2__10 mV/s, SnS_2__50 mV/s, SnS_2__100 mV/s and SnS_2__200 mV/s samples, respectively. The corresponding η_10_ values with iR compensation are found to be 73.8, 39, 44.3 and 93.8 mV. As shown in Fig. 5B, the Tafel slopes for samples SnS_2__10 mV/s, SnS_2__50 mV/s, SnS_2__100 mV/s and SnS_2__200 mV/s are 56 mV, 58 mV, 55 mV and 62 mV/dec respectively, suggesting the Volmer–Heyrowsky mechanism^27^. The mass activity of EADD synthesized samples show 14 times better performance than Pt/C, as illustrated in Fig. 5C. Electrochemical impedance spectroscopy was used to evaluate internal resistance (R_iR_) and charge transfer resistance (R_ct_), which provided R_iR_ values of 2.64 Ω, 3.82 Ω, 4.80 Ω, 3.87 Ω and 2.52 Ω and charge R_ct_ values of 148 Ω, 11.37 Ω, 9.56 Ω, 6.63 Ω and 16.79 Ω for SnS_2__NA, SnS_2__10 mV/s, SnS_2__50 mV/s, SnS_2__100 mV/s and SnS_2__200 mV/s, respectively as shown in Fig. 5D. This indicates the faster electron transfer rate on Pt deposited samples leading to efficient HER kinetics. Accelerated stability test (AST) conducted at 100 mV/s for sample SnS_2__50 mV/s showed stable performance of the catalyst at 10 mA/cm^2^, 20 mA/cm^2^ and 50 mA/cm^2^ for 28 h as shown in Fig. 5E. After AST, LSV was also conducted to evaluate the polarization performance, which is found to be comparable to LSV prior to AST, as shown in Fig. 5F. The XPS spectrum (shown in Fig. S6) revealed Pt in elemental state justifying the presence of particle agglomeration of Pt atoms in the reduction potential corresponding to HER.

**Figure 6:**
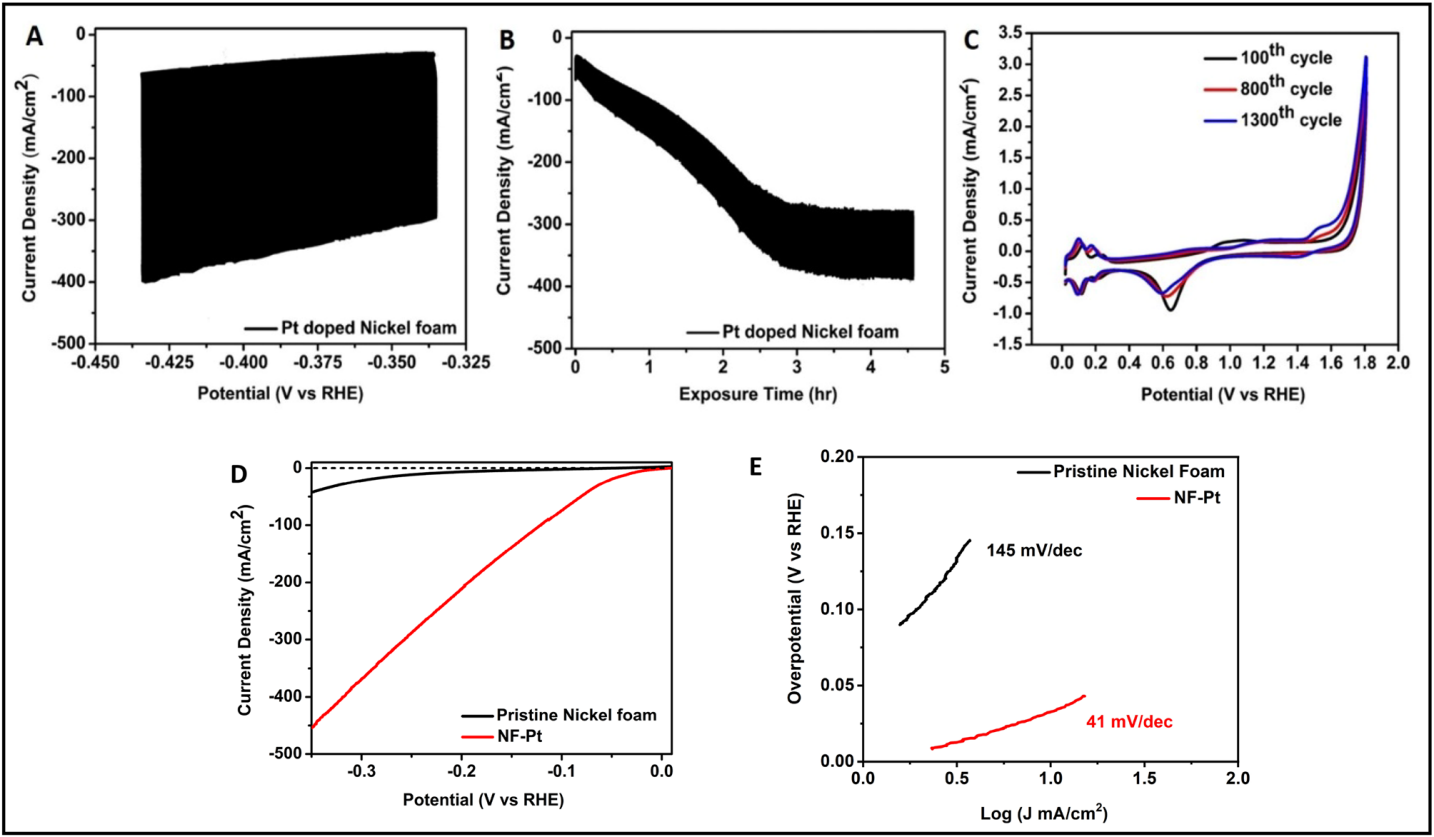
Shows the EADD synthesis for Pt decorated nickel foam and the corresponding HER performance of the synthesized catalyst in 0.5 M H_2_SO_4_ aqueous solution. (**A**) Shows the CV scans performed over nickel foam as anchoring substrate electrode. (**B**) Shows the J vs t plots of ASE during EADD of 4.5 h, (**C**) shows the CV scans of Pt sacrificial electrode at three different cycles. (**D**) Shows the polarization curves taken at 2 mV/s in an acidic medium. (**E**) shows the Tafel plots.

Although, the defect-rich SnS_2_ nanosheets decorated with trace amounts of Pt via dissolution-deposition process employing a three electrode assembly exhibits improved HER performance. However, during EADD synthesis in three-electrode assembly, the electrochemical cell provides uncontrolled potential at CE with very high scan rates, degrading dissolution kinetics and resulting in low efficiency of Pt electrodeposition on WE. After revealing limitations of the three-electrode assembly for the enrichment of HER catalytic activity of SnS_2_ by atomically deposited Pt using the EADD process, the use of the four-electrode assembly is demonstrated for the first time in this work. To the best of our knowledge, 2D SnS_2_ has been sparsely studied as catalyst materials for HER. Therefore, we decided first to perform bi-potentiodynamically controlled HER experiments using well-known electrocatalyst materials for HER viz, Ni foam^28,29^ to better understand the EADD process. In this experiment, nickel foam is used as anchoring substrate (WE_1_), polycrystalline (Pt) as a sacrificial electrode (WE_2_) with one common Pt (CE) and Ag/AgCl as the reference electrode. It has been demonstrated earlier that dual independent cyclic voltammetry renders electrochemical control over two electrodes and allows mechanistic evaluation of electrochemical reactions^30^. In a bi-potentiodynamically controlled four-electrode assembly (FEA), the dissolution of Pt (used as a sacrificial electrode, WE_2_) can be easily controlled by deploying another WE_1_, as shown in Fig. 1B. This arrangement provides better control of Pt dissolution kinetics and hence enables controlled deposition of Pt over anchoring substrate electrode (ASE). Porous Ni foam has often been employed as one of the active catalysts for HER in its pristine form^31^. However, in an acidic medium, its performance deteriorates with time due to oxide formation^32^. Decorating Ni foam with noble metal (Pt) in ultra-low amounts can significantly improve its catalytic activity as well as stability^29^. Hence, we have attempted to atomically decorate Ni foam with Pt using a controlled bi-potentiodynamic EADD process. The bi-potentiodynamic EADD process is deployed in a narrow potential range of − 0.435 V_RHE_ to − 0.335 V_RHE_ at 50 mV/s for 4.5 h (~ 4050 cycles), so that its oxidation and thereby dissolution can be prevented. Right from the beginning, CV scans over the chosen potential window showed significant HER activity over Nickel foam (WE_1_). While WE_2_, Pt, is deployed in 0–1.8 V_RHE_ at 300 mV/s for 4.5 h (1350 cycles)) to encounter optimum dissolution-deposition kinetics. The J vs. V plot is shown in Fig. 6A, while Fig. 6B represents the corresponding J vs. t plot. It is observed that Pt deposition starts since the very first cycle of the EADD process and an increment in HER current is noted until the 3rd hour which then saturates. These results corroborate the present findings over SnS_2_, stating that the controlled EADD process promotes Pt deposition up to a certain extent and then impedes its further deposition. Moreover, with the increasing cycles, there is a negative shift in oxide reduction peak of Pt in the voltammogram, suggesting induced irreversibility due to oxide formation (Fig. 6C). The potential on the common Pt_CE_ is also measured during the synthesis, with lower potential at CE (E_L/CE_) increasing from 2.06 to 2.54 V_RHE_ while upper potential at CE (E_U/CE_) increasing from 2.13 to 2.63 V_RHE_ which suggests the growth of oxide layer over Pt­ CE surface thus passivating dissolution. The prepared catalyst (Pt decorated Ni foam) was used to evaluate the HER performance in a conventional three-electrode cell assembly in an acidic medium (0.5 H_2_SO_4_). As shown in Fig. 6(D,E), the HER performance of the cell exhibit that Pt decorated Ni foam possesses η_0_ of ~ 0 mV (vs. RHE), η_10_ of 33 mV (vs. RHE), and a Tafel slope of 41 mV dec^−1^. Whereas the onset potential η_0_ of 78 mV (vs. RHE), the η_10_ of 239 mV (vs. RHE), and Tafel slope of 145 mV dec^−1^ are recorded for the pristine Ni foam. Furthermore, the prepared catalyst was also used to evaluate the HER performance in N_2_ purged 1 M KOH as a test electrolyte to validate its enhanced catalytic. Supplementary Fig. S7 shows the polarization curves of Pt decorated Ni foam and pristine Ni foam and with η_0_ of 0 mV and 150 mV while η_10_ of 42 mV and 237 mV, respectively. Taking iR compensation into account, the Pt decorated Ni foam shows a lower value of η_10_ = 34 mV. Furthermore, Tafel plots showed a significant improvement from 151 to 52 mV/dec. Moreover, the mass activity of 150 mA/cm^2^·mg at 33 mV_RHE_ is observed, which is 15 times higher than Pt/C. Nyquist plots obtained using EIS at − 0.1 V_RHE_ also corroborate the polarization results with R_iR_ of 0.95 Ω and 0.94 Ω and R_ct_ of 90.02 Ω and 6.11 Ω for pristine Ni foam and Pt decorated Ni foam respectively. SEM-EDS elemental mapping of Pt doped Ni foam is shown in Fig. S8, Fig. S9, Table S2 and Table S3 (in Supplementary Information), where the uniform distribution (~ 1 wt%) of Pt is readily observed.

## Conclusions

We have explored the influence of potential scan rates at the working electrode on the dissolution and deposition phenomena of Pt from the counter electrode in the EADD synthesis process. In a three-electrode assembly, when the working electrode is swept at 50 mV/s, the Pt counter electrode encounter scan rates of 1000–3000 mV/s, resulting in uncontrolled dissolution-deposition kinetics. Furthermore, a new insight into Pt dissolution-deposition behavior is presented to better understand and manage the EADD process for deposition of noble metal catalysts at the atomistic level. For the first time, four-electrode assembly using a bi-potentiostat is deployed to make the EADD synthesis relatively pragmatic, achieve better control and improve the efficiency of the Pt dissolution-deposition process. The present work also reflects that the bi-potentiodynamic EADD technique to be a potentially versatile surface synthesis method for atomistic deposition of Pt to improve catalytic activity and stability of HER catalysts. Herein, we deliver that EADD is seemingly not limited to Pt deposition, moreover, it can be extended to other transition elements (E.g. Ni, Co, Pd, Fe, etc.) to have a controlled electrodissolution from metal electrodes and simultaneous electrodeposition on various conductive supports,  e.g., Ni foam used in the present work.

### Supplementary Information

Supplementary Information.

## Data Availability

The datasets used and/or analysed during the current study available from the corresponding author on reasonable request.
