# Supplementary material for: Mechanistic insights on Bi-potentiodynamic control towards atomistic synthesis of electrocatalysts for hydrogen evolution reaction
Source: Sci Rep. 2023 Sep 30;13:16433. doi: 10.1038/s41598-023-43301-9 (PMC10542813; doi:10.1038/s41598-023-43301-9)
Supplement: Supplementary file 1 — Supplementary Information. [file 41598_2023_43301_MOESM1_ESM.docx]

**Supporting Information**

**Mechanistic Insights on Bi-Potentiodynamic Control towards Atomistic Synthesis of Electrocatalysts for Hydrogen Evolution Reaction**

*Rohit Ranjan Srivastava^1^, Divyansh Gautam^2^, Rajib Sahu^3^, P.K Shukla^4^, Bratindranath Mukherjee^2^, Anchal Srivastava^1*^*

^1^ Department of Physics, Institute of Science, Banaras Hindu University, Varanasi-221005

^2^ Department of Metallurgical Engineering, Indian Institute of Technology-BHU, Varanasi-221005

^3^ Max-Planck-Institut für Eisenforschung, Düsseldorf-40237, Germany

^4^Vindhya Institute of Technology and Science, Satna (MP)-485001, India

* Corresponding Author

*Email:* anchalbhu@gmail.com, anchal@bhu.ac.in,


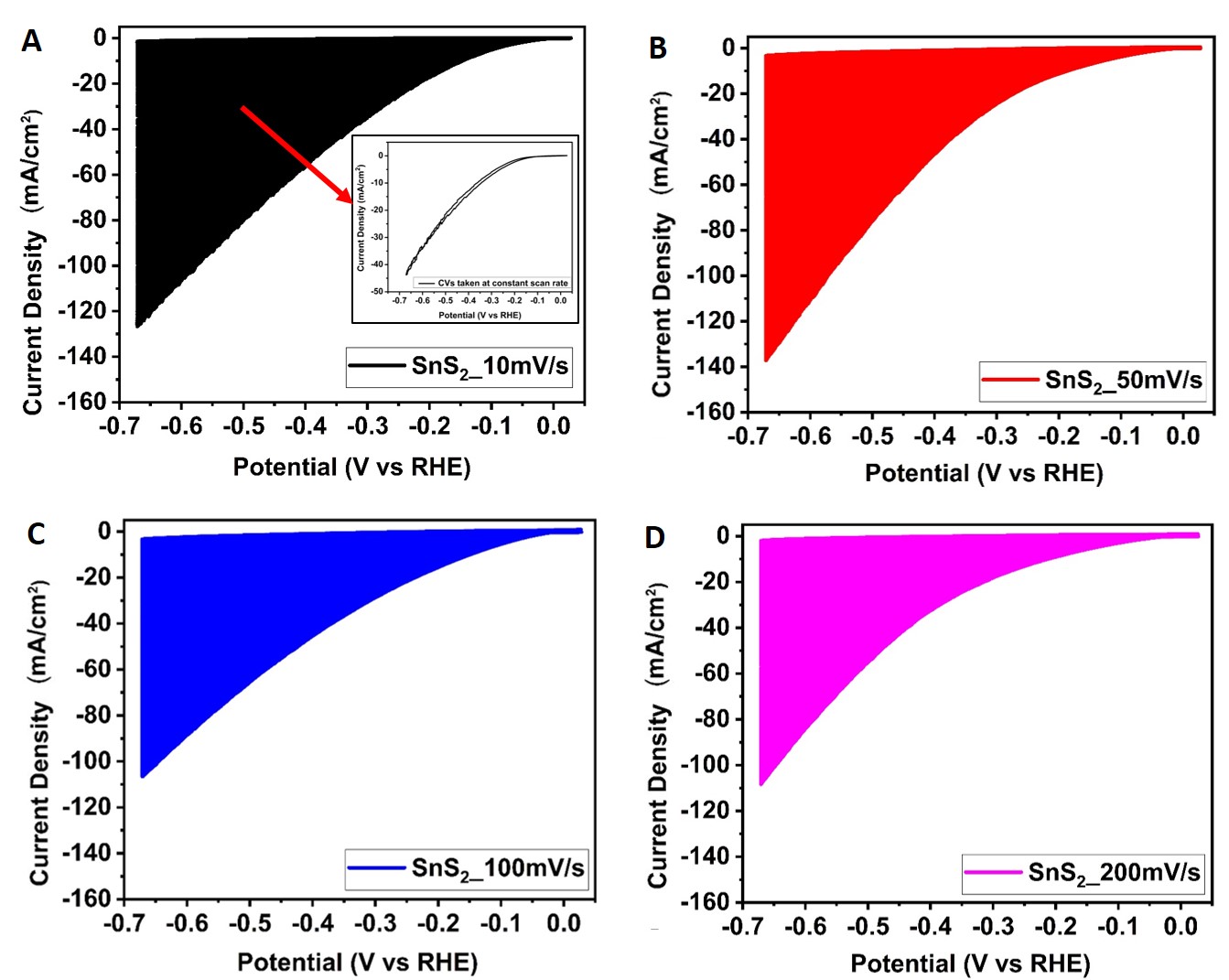


**Figure S1**: CV Scan at scan rates of **(A)** 10mV/s, **(B)** 50mV/s, **(C)** 100mV/s and **(D)** 200mV/s during EADD synthesis SnS_2_ (WE)

*
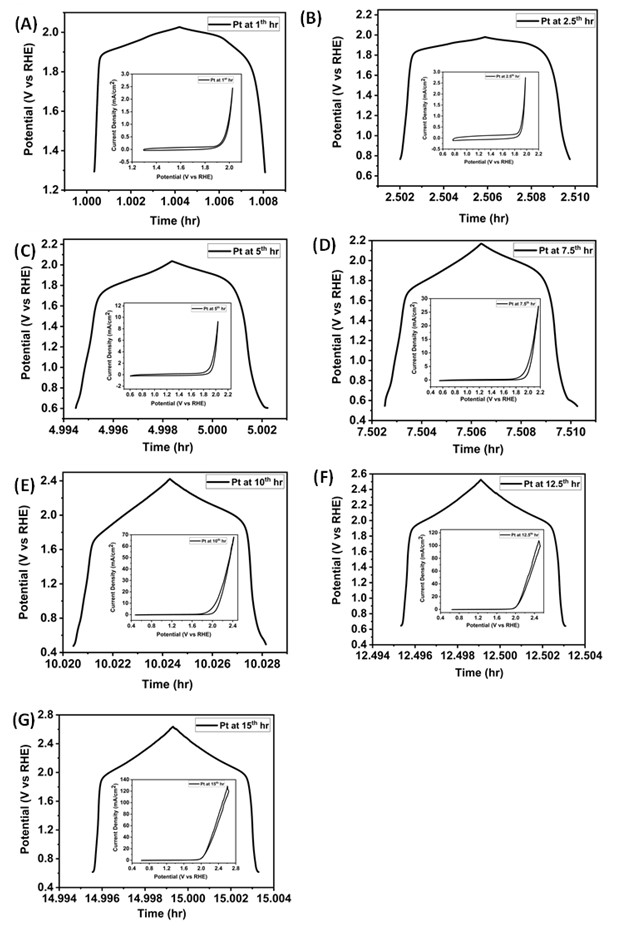
*

**Figure S2:** shows potential vs time plots over Pt (as SE/CE) for 7 different cycles at **(A)** 1^st^ hr, **(B)** 2.5^th^  hr **(C)** 5^th^ hr **(D)** 7.5^th^ hr **(E)** 10^th^ hr **(F)** 12.5^th^ hr and **(G)** 15^th^ hr over SnS_2_ (as ASE/WE) at a constant scan rate of 50 mV/s for 2000 cycles (~15 hr). Insets in each figure from **(A) to (G**) shows the current density (J) vs Potential (V vs RHE) over Pt for the corresponding cycles.

| **Time (in hour)** | **Scan Rate V_C_ (V/s)** |
| --- | --- |
| 1 | 1.0 |
| 2.5 | 1.3 |
| 5 | 1.1 |
| 7.5 | 1.4 |
| 10 | 2.0 |
| 12.5 | 2.5 |
| 15 | 2.9 |

**Table S1**: shows the cathodic scan rate (V/s) over Pt_CE_


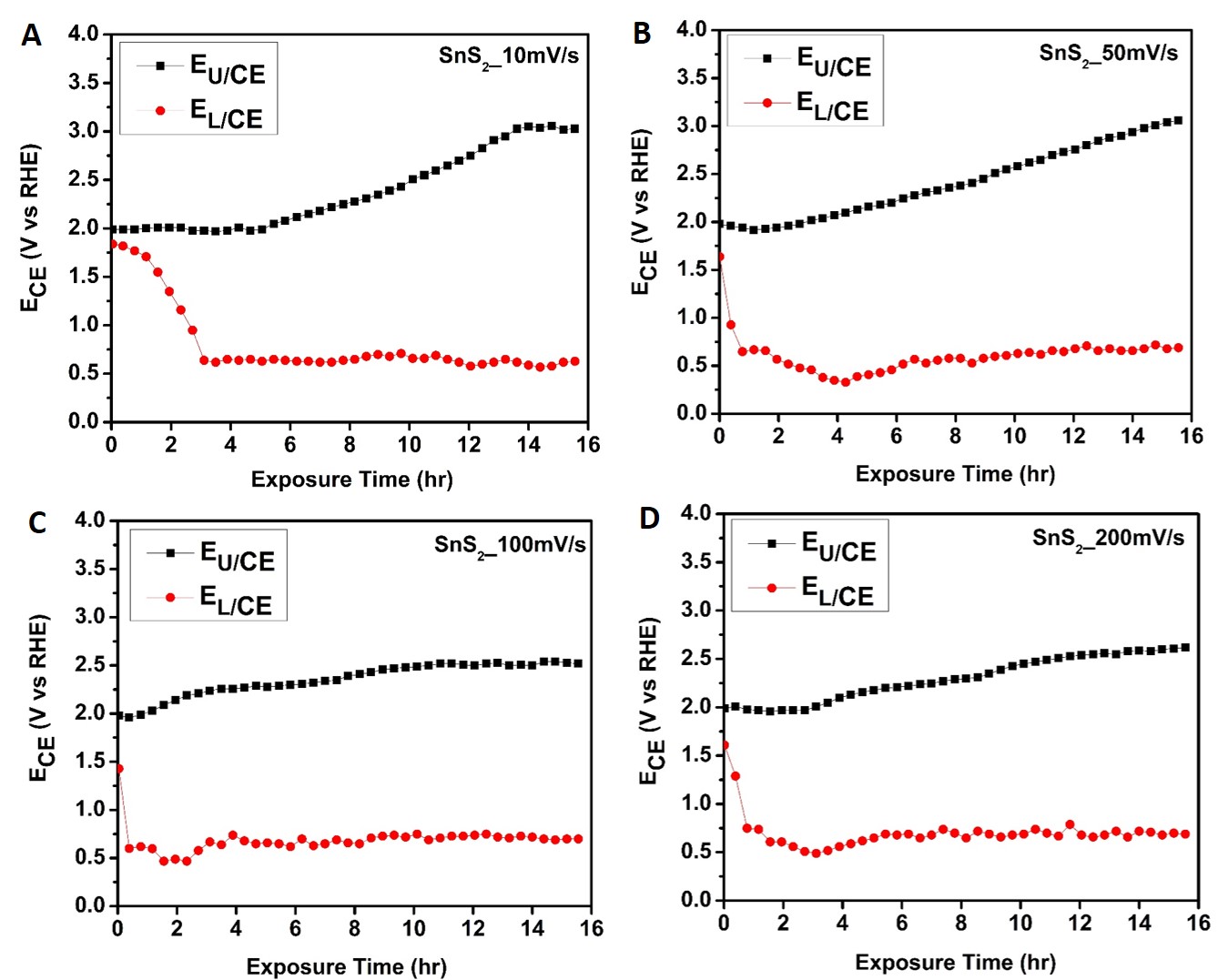


**Figure S3:** Potential on Pt sacrificial counter electrode (E_CE_) at different time interval during EADD experiment for different scan rates **(A)** 10mV/s **(B)** 50mV/s **(C)** 100mV/s and **(D)** 200mV/s. E_U/CE_ and E_L/CE_ is the upper and the lower limit of the potential on Pt sacrificial/counter electrode during a particular CV scan.


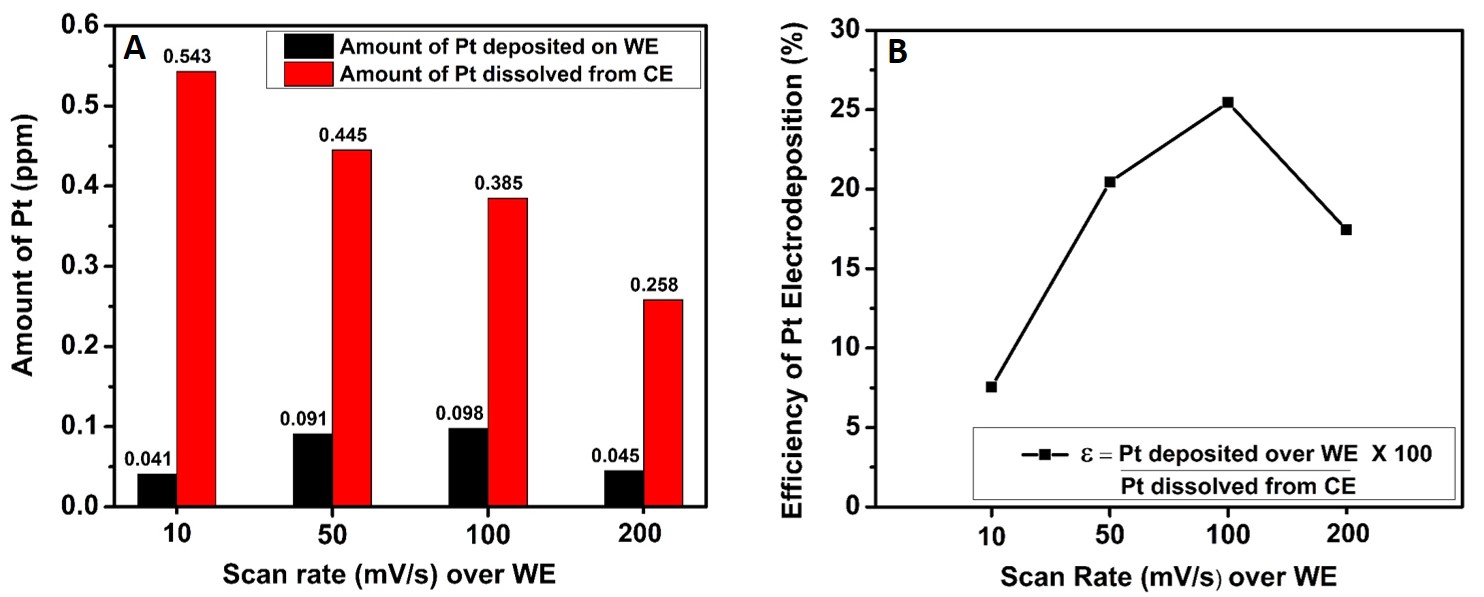


**Figure S4:** **(A)** Amount of Pt (in ppm) dissolved in electrolyte (Red bar) from counter electrode (Pt) after ~15 hr of EADD synthesis at various scan rates over SnS_2_ (WE). Amount of Pt (ppm) deposited (Black bar) over SnS_2_ (WE) from the Pt ions present in the electrolyte. **(B)** shows the efficiency of Pt deposition over SnS_2_ with varying scan rate over WE.


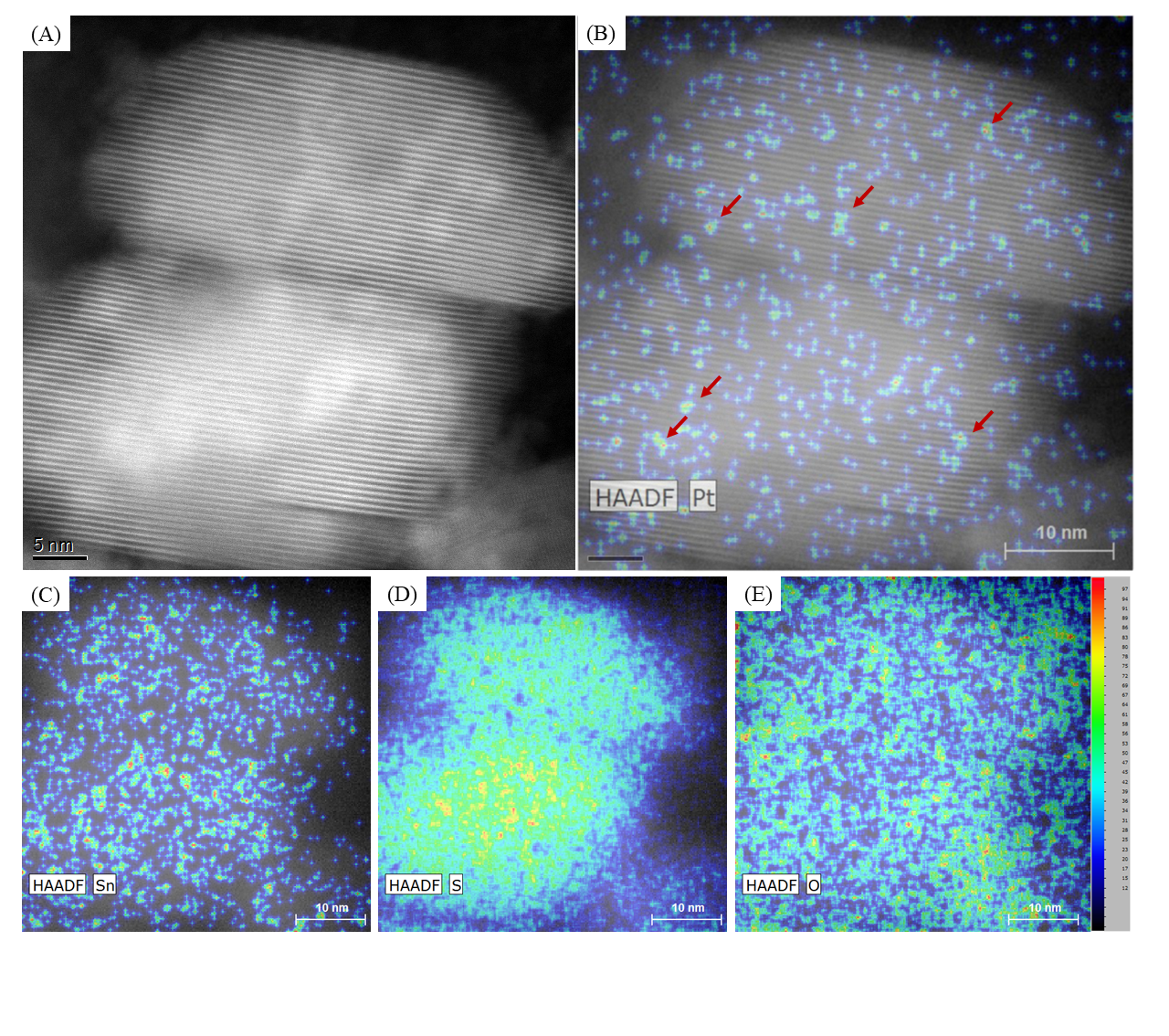


**Figure S5:** **(A)** STEM micrograph of rolled surface of Pt deposited SnS_2_ and **(B)- (E)** shows relative elemental intensity distribution map of Pt, Sn, S and O respectively. Representative Pt nano-clusters are marked by red arrows in **(B).**


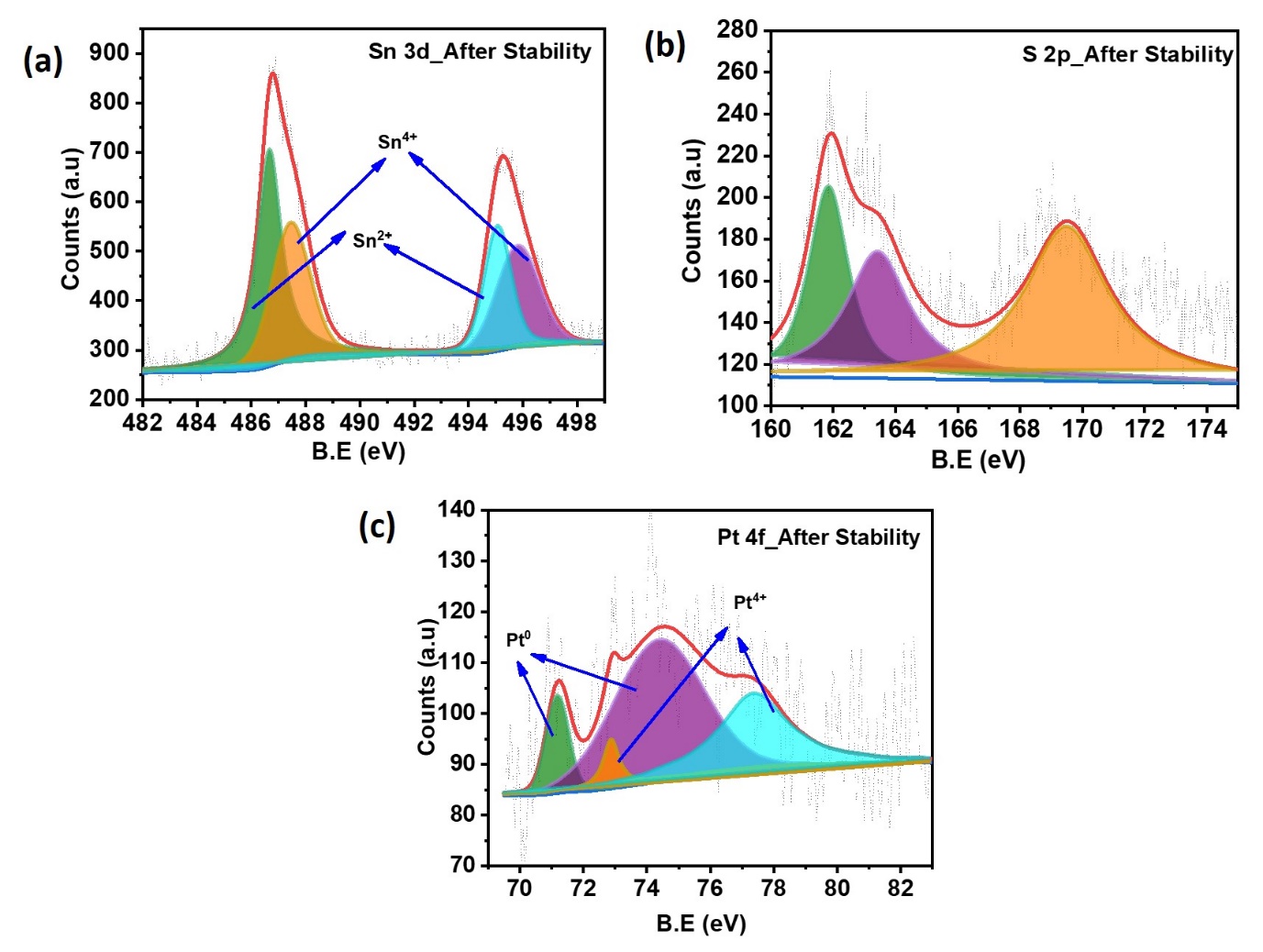


**Figure S6:** XPS spectra of SnS_2__50mV/s after accelerated stability test of 28 hr.

**

**Figure S7:** Shows the EADD synthesis for deposition of Pt over nickel foam and corresponding HER performance of the synthesized catalyst in 1M KOH aqueous solution. **(A)** shows the polarization curves taken at 2mV/s. **(B)** shows the Tafel plots. **(C)** shows corresponding Nyquist plots from EIS measurement.


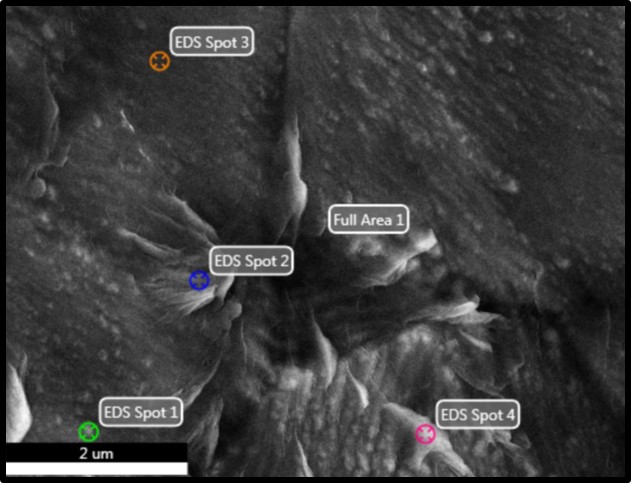


**Figure S8 :** Shows the TEM micrograh of Pt decorated Ni foam prepared by four-electrode assembly.


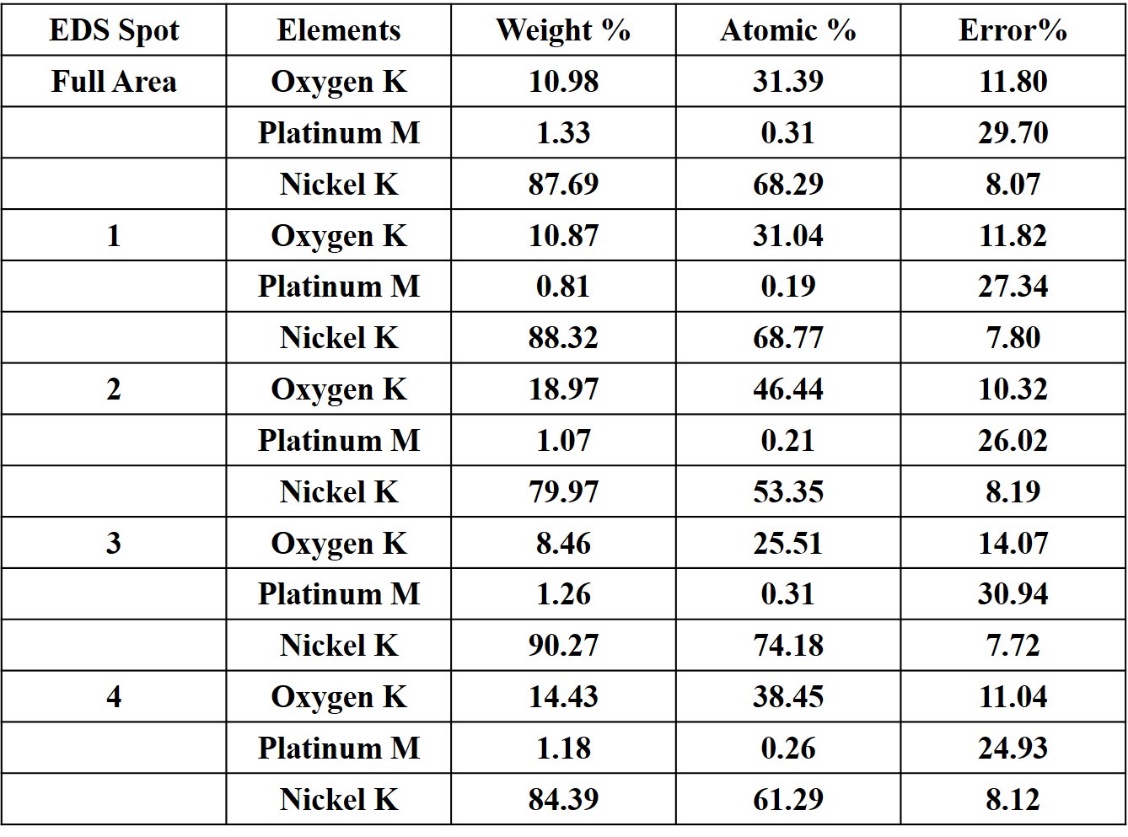


**Table S2 :** shows the distribution of elements (EDS data) obtained from the regions marked on the micrograph of Figure S8.


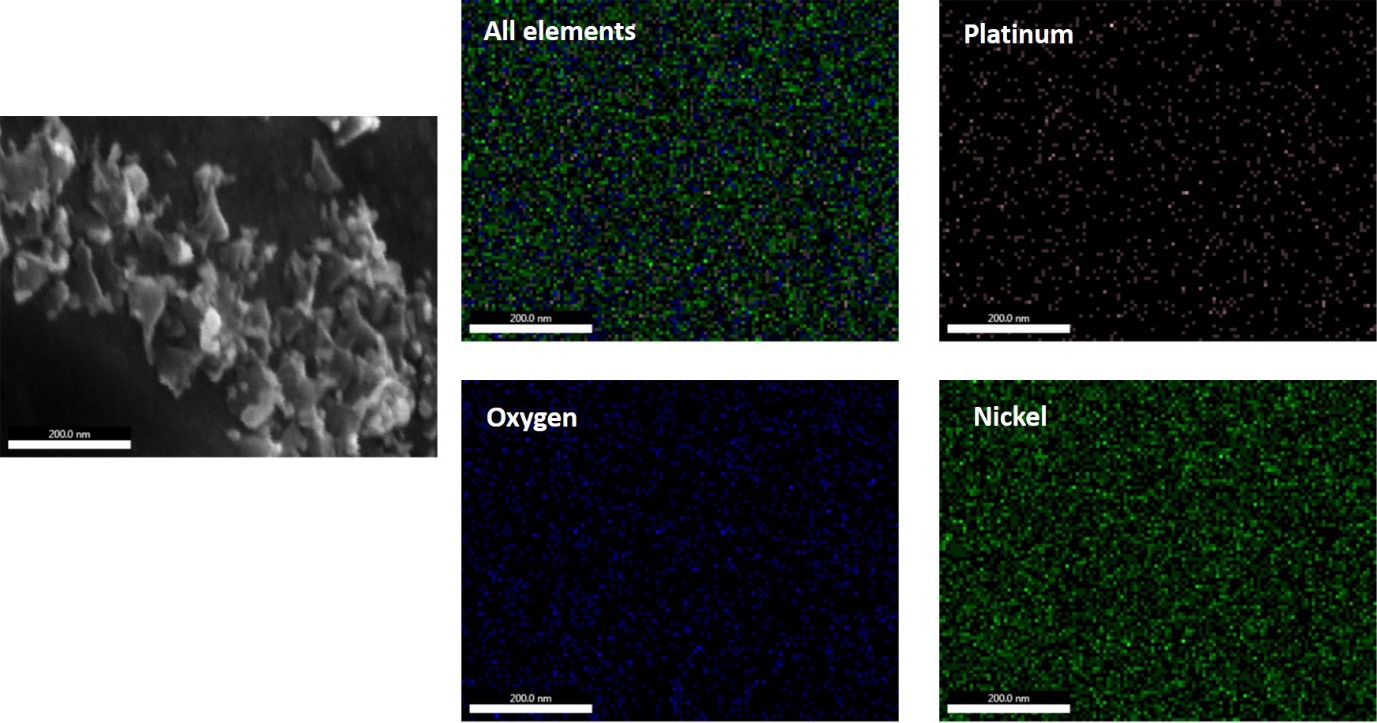


**Figure S9 :** Shows the EDS mapping of Pt decorated Ni foam prepared by four-electrode assembly.


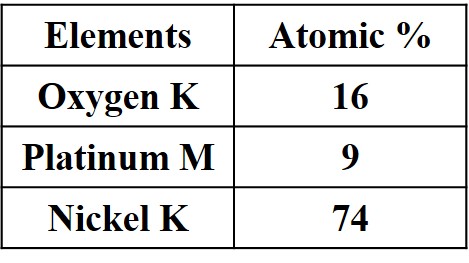


**Table S3 :** shows the distribution of elements (EDS data) obtained from the micrograph of Figure S9.
